# Impact of hydropower availability on resource adequacy of the United States western interconnection

**DOI:** 10.1371/journal.pone.0351321

**Published:** 2026-06-23

**Authors:** Sai Veena Sunkara, Srihari Sundar, Gord Stephen, Stuart Cohen, Patrick Reed, Vivek Srikrishnan, Ariel Miara

**Affiliations:** 1 School of Civil and Environmental Engineering, Cornell University, Ithaca, New York, United States of America; 2 Grid Planning and Analysis Center, National Laboratory of the Rockies, Golden, Colorado, United States of America; 3 Department of Biological and Environmental Engineering, Cornell University, Ithaca, New York, United States of America; Technical University of Berlin: Technische Universitat Berlin, GERMANY

## Abstract

Hydropower is a key energy source in the western interconnection of the United States, comprising about 25% of the annual installed nameplate capacity in 2020. However, its generation is increasingly impacted by changing hydrological conditions, operational constraints, environmental factors, water availability, and aging dam infrastructure. Assessing hydropower availability and resource adequacy is valuable for shaping energy policies and infrastructure requirements. In this study we evaluate the sensitivity of resource adequacy in the Western Interconnection to the unavailability of hydropower plants, under a wide-ranging set of 16,384 hydropower loss scenarios derived from a combination of 14 major hydrologic regions. Although a complete loss of hydropower capacity in any region is unlikely, studying such scenarios helps in identifying hydrologic regions most critical for maintaining resource adequacy. So, we identify key hydrologic regions with disproportionately large adequacy impacts relative to their installed hydropower capacity and study compounding effects between regions using classification and regression trees. Even after controlling for total installed capacity, we find that hydropower resources in the Pacific Northwest contribute the most to interconnection-wide adequacy outcomes, with resources in Northern California and the Desert Southwest providing more moderate incremental contributions.

## 1 Introduction

Hydropower plays a key role in global electricity generation, representing 16% of the world’s total electricity production and 41.8% of global renewable capacity in 2021 [1,2]. In addition to being a source of renewable electricity, hydropower has many economic benefits, including low operating costs, high efficiency, high flexibility, and long infrastructure lifespans [3–5]. Hydropower offers many other benefits as well, including managed water supply, flood control, navigation, and recreation [6,7].

As a dispatchable, large-scale renewable energy resource, hydropower supports long-term grid resilience and energy security. In the United States (US), hydropower plays a crucial role in the energy mix, including meeting approximately 25% of the electricity demand in the Western Interconnection of North America [8]. The Western Interconnection encompasses a vast and geographically diverse region spanning 14 US states, two Canadian provinces, and the Baja California peninsula in Mexico. Hydropower plays a crucial role in this system, providing consistent low-marginal-cost electricity, balancing grid variability, and supporting grid stability [9].

Although hydropower is expected to remain a key component of global power systems, its generating potential faces dynamic and growing challenges. Climate and weather variability are leading to changes in precipitation patterns, rising temperatures, and a range of challenging extreme events. These evolving extremes in combination with aging infrastructure systems are challenging hydropower plants to provide consistent energy output while meeting other power sector and water management needs [10,11]. Competing water demands for agriculture, urban consumption, and ecosystem sustainability further constrain hydropower generation, as these needs typically take priority over power system needs [12]. Structural, operational, and ecological challenges, such as barriers to fish migration, sedimentation, decreasing flood control capacity, changes to reservoir management policies, environmental regulations, and market developments, all play a critical role in determining how hydropower can and will operate [13–15]. Aging infrastructure and deferred maintenance of many dams introduce additional challenges, such as high costs, safety concerns, and reduced efficiency, which may require costly upgrades or sometimes decommissioning [16,17]. In some cases, dam removal for river restoration, ecosystem recovery, and public safety considerations contributes to the uncertainty surrounding future hydropower availability [14,15]. While many of these drivers typically result in partial, seasonal, or policy-driven reductions in hydropower output, they also point to the possibility of sustained or permanent capacity withdrawal under more extreme structural or regulatory outcomes. These considerations demonstrate the importance of assessing how reduced hydropower availability impacts power system reliability.

Recent studies have explored changes to hydropower energy availability, including extreme weather impacts of water availability and how it affects grid operational performance and economics [18,19]. Additionally, recent studies have explored the effects of sediment control strategies and mitigating barriers to fish migration [20,21]. However, these studies do not adequately inform the challenges that emerge as the Western Interconnection navigates grid supply and demand transitions, where understanding the interplay between hydropower availability and system reliability is essential for shaping robust energy policies and infrastructure investment strategies. A better understanding of the interplay of hydropower availability and system reliability offers significant value for guiding the optimal integration of other renewable energy sources, such as solar and wind limit system reliability risks [22].

A key challenge in the literature on hydropower availability is evaluating the power system reliability consequences that arise when dams/power plants are unavailable [23]. Here, we focus on the resource adequacy component of system reliability to understand hydropower contributions during periods of grid stress when available capacity may be unable to meet demand. Resource adequacy describes the bulk power system’s ability to balance supply and demand with an acceptably low risk of supply shortfall in all time periods and locations on the grid [24]. Recent research has emphasized the critical need to quantify resource adequacy for different energy resources (as biomass, wind and solar), enabling more robust assessments of system reliability [25,26]. Previous work assessing how hydropower impacts resource adequacy has explored water availability impacts from both changes in environmental conditions and load growth [27]. However, the impact of uncertain and concurrent hydropower unavailability across multiple regions of an interconnected power system such as the Western Interconnection has not been studied, and the critical hydropower regions that drive system reliability are not necessarily clear from first-order observations such as hydropower generation or capacity share. In this work, we study the resource adequacy impacts of hydropower unavailability throughout the Western Interconnection as well as the extent to which these impacts interact spatially and compound across the electrical system.

Given the broad range of uncertain factors affecting hydropower generation, it is important to rigorously explore how hydropower availability influences system performance, resource adequacy, and energy reliability. In this study, we assess the sensitivity of the US footprint of the Western Interconnection to the availability of hydropower generation, resolved to 14 regional hydrologic basins. We use exploratory modeling to analyze an ensemble of 16,384 scenarios to uncover conditions where hydropower shortages in a basin lead to substantial changes in resource adequacy. Exploratory modeling is widely used in the literature to systematically evaluate scenario ensembles and discover important vulnerabilities and the factors that cause them [28,29]. While the influence of meteorological drivers on the performance and vulnerabilities of renewable energy systems has been the focus of prior work [30,31], this study’s explicit accounting of the interdependent effects of changes in regional hydropower availability provides important insights for better mapping potential sources of system reliability vulnerabilities.

The systematic evaluation of a range of possible operating contexts makes this approach different from traditional scenario-based modeling where the key factors are specified in advance. Instead, we focus on “scenario discovery” using broader ensembles of results to better understand the drivers of the most consequential outcomes [32,33]. The identification of consequential scenarios of interest is a central part of exploratory modeling [34,35]. Exploratory modeling and scenario discovery are reflective of a broader body of work related to decision-making under deep uncertainty [36]. Here, exploratory modeling for different scenarios of hydropower unavailability is performed to discover key hydropower assets and regions with outsized impacts on resource adequacy. Overall, our exploratory modeling analysis of the Western Interconnection aids in understanding the system’s vulnerability to reduced hydropower availability, both locally and across the broader system. It also represents a unique application of this approach with a complex, applied interconnect-scale grid model, presenting an approach that could be extended to additional or integrated energy system models.

Our exploratory modeling analysis elucidates the regional dependencies of hydropower generating basins and the broader effects of hydropower availability on resource adequacy. The exploratory analysis identifies key regions that significantly affect resource adequacy metrics and analyzes the spatial interactions between the regional hydrologic basins under alternative scenarios of hydropower availability. A complete loss of hydropower represents a deliberately conservative upper-bound case rather than a likely outcome. In practice, hydropower reductions would more plausibly occur through partial capacity reductions, seasonal water constraints, or multi-year droughts. Including this extreme case therefore brackets the range of possible system responses and provides a clear benchmark for understanding hydropower’s role in adequacy. Within this analysis, the resource adequacy framework does not track temporal interdependencies across months and therefore cannot explicitly represent reservoir management or the intertemporal shifting of generation across periods. As a result, hydropower availability is represented through binary capacity removal with fixed monthly energy budgets for the remaining resources, which prevents generation during constrained periods from being reallocated to other times of the year. However, hydropower designated as dispatchable can shift energy within a given monthly allocation to follow load and other system needs within that month, and this degree of flexibility is represented in the analysis. The inability of remaining hydropower resources to change monthly energy allocations in response to hydropower unavailability scenarios represents a conservative stress test from a power sector perspective, providing an upper-bound estimate of potential reliability impacts. This approach helps quantify the magnitude of hydropower’s adequacy contribution when generation is constrained and highlights how other resources such as thermal generation, storage, imports, and demand-side flexibility would need to compensate. It also reveals interconnection dependencies and potential adequacy vulnerabilities that may not be apparent under baseline assumptions.

## 2 Methods

**Fig 1:**
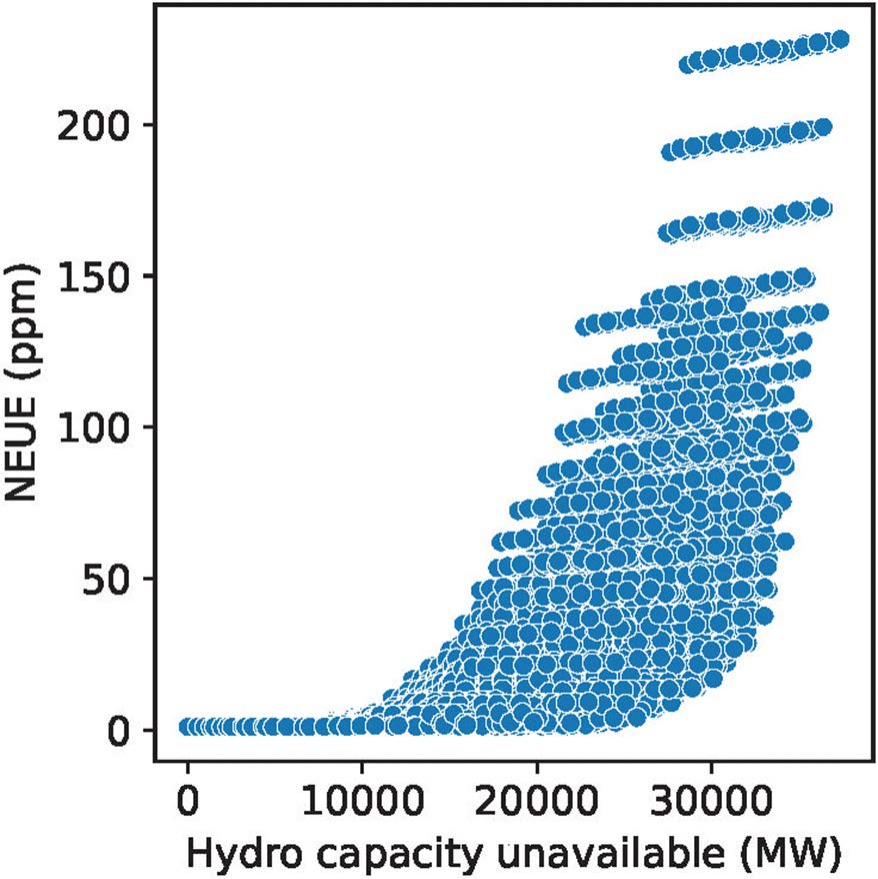
Analytical framework for quantifying resource adequacy impacts of reduced hydropower availability, including identifying regions with outsized adequacy impacts relative to installed hydropower capacity.

Our analytical framework, visualized in Fig 1, starts with using a capacity expansion model to simulate a base fleet to determine electrical generation and transmission infrastructure for use in resource adequacy modeling (Section 2.1). We then model hydropower unavailability by enumerating different combinations of plant removals across regional basins (Section 2.2). We modify our base fleet for each of these scenarios by eliminating capacity from the corresponding plants, then we calculate system resource adequacy metrics for that scenario (Section 2.3). Finally, we use the resource adequacy results from all scenarios to find the most consequential hydrological regions using scenario discovery (Section 2.4).

### 2.1 Baseline infrastructure portfolio

We use the National Laboratory of the Rockies (NLR)’s Regional Energy Deployment System (ReEDS™) capacity expansion model [37,38] to identify a least-cost generation portfolio representing a potential baseline grid condition for the Western Interconnection in the year 2050. ReEDS is used to conduct long-term grid planning research and analysis for the US. It is a large-scale linear program that minimizes the net present value of investment and operation costs for the contiguous US grid in each of a set of predefined model years. The year 2050 was chosen for this work because it represents a long-term planning horizon over which hydropower availability is subject to deep uncertainties. ReEDS requires the least-cost system to meet demand for electricity, capacity, and ancillary services across 35 regions in the Western Interconnection, considering factors such as capital and operating costs, policy constraints, and 7 years of historical weather data (2007–2013), which influence electricity demand and generation from variable renewable energy sources. Information on regional demand patterns with solar and wind output derived from historical weather can be found in supplementary S5–S7 Figs. Hydropower availability is represented using seasonal capacity factors calculated for each existing plant based on historical generation data from 2007–2013. Reservoir hydropower is not subject to explicit ramping constraints; operational limits are represented through seasonal variation in turbine head, which affects maximum hourly dispatchable generation, together with fixed monthly energy budgets. Pumped hydro storage resources are included in the system and remain fully available in all hydropower unavailability scenarios. We use the Mid Case electricity growth assumptions from the 2023 ReEDS Standard Scenarios [39], which comprise default or reference data and assumptions that include current enacted policies at the time of publication (Policies in this scenario include the tax incentives for wind, solar, hydropower, and other technologies enacted under the 2022 Inflation Reduction Act. Implications of more recent policy changes are discussed in the Discussion and Conclusion section) and central projections of load growth and technology change. This version of ReEDS uses planning reserve margins to ensure sufficient supply is procured in different regions of the system, and we iteratively tune these reliability parameters to achieve a system that meets typical accepted resource adequacy criteria in each of the Western Interconnection’s three reserve sharing regions (Section 2.3 provides further discussion of such criteria).

In the capacity expansion model (ReEDS), we use a single generation and transmission capacity layout that serves as the common baseline for all scenarios. Hydropower unavailability is introduced only in the resource adequacy stage, so the analysis evaluates how this fixed system performs under different hydropower unavailability conditions rather than adjusting capacity to mitigate them.

### 2.2 Hydropower unavailability scenarios

Our representation of the Western Interconnection includes hydropower plants with a total nameplate capacity of approximately 55 GW, which includes existing as well as new hydropower plants. We consider the unavailability of currently operational plants which exist in our target fleet. Studying all possible combinations of plant unavailability would be computationally intractable. Instead, we prescribe unavailability by considering 14 geographically proximate groups of plants based on their shared basin and consider the loss of capacity from entire basins at once. We assume full removal of hydropower nameplate capacity for the entire resource adequacy assessment period of 2007–2013, which is used to encompass uncertain weather related components included in the analysis (load, variable generation, and hydropower). We acknowledge that complete loss of all hydropower capacity in a given basin is not likely or practical; however, studying impacts in this way enables computational tractability and signals which groups of hydropower resources are most important for maintaining resource adequacy.

**Fig 2:**
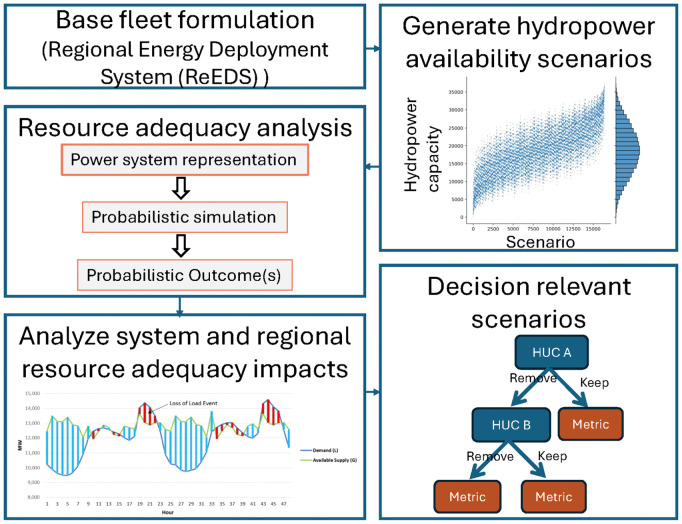
Hydrologic regions considered in the scenario generation process. The inset depicts the three subbasins considered within the Upper Columbia region. Regions are colored according to their total nameplate hydropower capacity, and green circles represent the location of hydropower plants considered in the analysis, with size proportional to their capacity.

Plants are grouped using the US Geological Survey hydrological unit code (HUC) regions they belong to. The HUC classification is a hierarchical system used to identify drainage basins at specific spatial scales. Each HUC represents a specific geographic area within a watershed and provides a spatially consistent framework for grouping multiple hydropower plants within the same regional hydrologic basin to analyze the collective impact of hydropower availability. HUC regions are delineated at different levels, where a two-digit designation, or HUC2, represents hydrologic regions, HUC4 represents hydrologic subregions, and HUC8 represents subbasins. We start by aggregating at the HUC4 level, considering all HUC4s that have a nameplate hydropower capacity of more than 500 MW. This yields 12 HUCs that are candidates for unavailability scenarios. Of these, the Upper Columbia (UC) subregion (HUC 1702) has a high concentration of large plants, so we split it further into its three HUC8 subbasins (Chief Joseph, Entiat, and Priest Rapids), resulting in 14 candidate removal regions (Fig 2). We explore all 2^14^ (16,384) possible discrete combinations of unavailability of these regions. S1 Fig of the supporting information shows the distribution of these scenarios as a function of unavailable nameplate capacity. These combinations produce scenarios with up to 37.4 GW of lost nameplate hydropower capacity, representing 68% of all hydropower capacity in the Western Interconnection. Table 1 lists the hydrological regions considered in the unavailability scenarios, with their corresponding HUC identifiers, installed hydropower nameplate capacities, and annual hydropower capacity factors (CF) which represents the aggregate annual energy capacity from the hydropower plants in each HUC.

**Table 1:** Hydrologic regions considered in generating hydropower unavailability scenarios.

Region Name	HUC ID	Hydropower Capacity (MW)	Annual Capacity Factors
Upper Colorado (CO)	1407	1316	0.29
Lower Colorado (CO)	1503	765	0.15
Kootenai-Pend Orielle-Spokane (KPOS)	1701	3175	0.24
Upper Columbia (UC) Chief Joseph	17020005	8495	0.35
Upper Columbia (UC) Entiat	17020010	2916	0.10
Upper Columbia (UC) Priest Rapids	17020016	961	0.07
Upper Snake	1704	574	0.14
Middle Snake	1705	1186	0.24
Lower Snake	1706	3793	0.13
Middle Columbia	1707	5916	0.12
Lower Columbia	1708	1184	0.09
Puget Sound	1711	996	0.06
Sacramento	1802	3944	0.01
San Joaquin	1804	2148	0.01
Total and percentage of US WECC hydropower capacity		37368 and 68%	

### 2.3 Resource adequacy assessment

We use NLR’s Probabilistic Resource Adequacy Suite (PRAS) to quantify the change in resource adequacy risk under each hydropower unavailability scenario. PRAS performs a fast screening analysis of a grid’s ability to balance supply and demand considering availability of generation, chronological storage dispatch, and interregional transmission flows [40]. In this case, we study each hydropower removal scenario independently in a Monte Carlo framework, considering 7 weather years of chronological electricity load and variable generation availability realizations alongside 1000 alternate realizations of thermal generator outage patterns. Individual hydropower plants are represented as either run-of-river (fixed hourly available capacity) or reservoir hydropower (with monthly energy budgets that can be dispatched more flexibly across time). No other aspects of the system (generation mix, transmission topology, fuel prices, etc.) were varied in this analysis other than hydropower capacity availability.

For each hydropower availability scenario, we calculate expected unserved energy (EUE) for the interconnection overall and for each electrical balancing area, aggregating results both seasonally and annually. EUE is a probabilistic adequacy risk metric reflecting the amount of electrical energy that the system failed to serve due to supply shortfalls or deliverability constraints, averaged across all probabilistic samples considered in the analysis [41]. This metric is also often reported in terms of normalized EUE (NEUE), the ratio of average unserved energy to total electrical energy demand. NEUE is reported in parts-per-million (ppm). Power systems using NEUE adequacy criteria today target values in the range of 10–20 ppm [41], although some studies suggest that values of 1–2 ppm may be more consistent with other accepted reliability standards moving forward [42]. The reserve margin calibration described previously in Section 2.1 was used to tune the base system to a 1 ppm NEUE criterion in each reserve sharing region of the Western Interconnection.

To contextualize the increase in system risk induced by the loss of hydropower resources relative to the size of those resources, we also calculate a capacity-normalized impact for each scenario by dividing the incremental EUE occurring in that scenario (energy risk beyond the baseline EUE) by the nameplate capacity of hydropower removed. The resulting metric, with units of MWh/MW, expresses the capacity-adjusted importance of different hydropower resources in mitigating grid shortfall risk and allows meaningful comparisons between plants and regions of varying sizes. This ΔEUE/MW metric can be calculated for a scenario on an average basis (considering all hydropower plants removed relative to the original base grid composition from ReEDS) or a marginal basis (considering the incremental impact of removing plants in a specific region, relative to a specified baseline).

### 2.4 Identification of critical hydrological regions

The electrical location of different resources on the grid, relative to other generation resources, transmission infrastructure, and demand, can cause differences in the consequences of unavailability, even after controlling for variations in the amount of installed capacity in each region. In this study, we use a scenario discovery approach to examine how availability of hydropower in specific hydrological regions influences system adequacy, and understand which regions are most influential in determining those outcomes, in terms of changes in EUE per megawatt of unavailable hydropower.

Scenario discovery is a data-driven analytical tool used to identify the most consequential factors or input parameters to a system in terms of impacts on outcomes of interest. This approach is widely used in the literature on decision-making under deep uncertainty and infrastructure planning [43–48]. The method includes identifying uncertainties, exploratory ensemble modeling to stress systems across combinations of the uncertainties, classifying the outcomes of interest based on performance metrics of interest, and applying machine learning methods or other statistical techniques to mine the large volume of output data and identify the consequential factors that drive key outcomes.

Classification and regression tree (CART) analysis is one of the most commonly used machine learning methods in scenario discovery, given that it can be applied to both binary and continuous data to identify relationships within complex datasets using a binary recursive partitioning method [49]. CART analysis uses human-interpretable decision trees to classify scenarios and identify input variables that trigger key outcomes. These approaches help find patterns in complex systems, focus on key uncertainties, and evaluate the system’s susceptibility to those uncertainties.

In this work, we carry out CART analysis with the scikit-learn Python package [50], using a maximum tree depth of 3 to balance interpretability and accuracy (An example CART tree with depth = 2 is shown in Fig 1). The model is trained on all regional unavailability scenarios, where the presence or absence of hydropower in each region serves as a binary input/feature variable (resulting in 14 feature variables overall). The change in system-wide EUE per megawatt of capacity removed is used as the response variable/classification criterion. The CART output consists of logical conditions that indicate which sets of regional hydropower unavailability provide the greatest predictive power in understanding system-wide, capacity-adjusted EUE impacts. A conventional scenario discovery approach identifies the influential input factors (in this case, availability of hydropower in specific regions) in a single step using CART analysis. Mathematically, the Gini index is the metric that measures how well the data is seperated at a node split in the CART tree [50]. But the partitions generated by the CART algorithm are inherently approximate, and as a result, certain data points may be assigned to incorrect classes. We tackle this misclassifications issue using a sequential screening approach by refining the dataset used to train the CART tree.

In this work, we apply a sequential screening process to systematically identify different regions that provide outsized impacts to system adequacy but may not be recognized with a traditional single-step CART analysis. We apply CART iteratively, screening scenarios at each step to identify the most important hydrological regions beyond those with the largest-capacity power plants. So, each step involves selecting the most important region identified by CART analysis and retaining scenarios where that region remains available for the next round of screening. The sequential screening process is performed iteratively to ensure that the most dominant regions do not overshadow the effects of less dominant but consequential regions impacting system EUE per megawatt of capacity removed.

While the CART analysis considers cumulative adequacy impacts across a set of regional hydropower unavailability, we also seek to understand the incremental impacts of availability in individual hydrologic regions and how those impacts may interact nonlinearly with hydropower availability in the rest of system. To study these dynamics, we compute the incremental increase in system-wide EUE per megawatt of capacity removed in a given region, including visualizing spatial patterns in these outcomes for specific sets of unavailabilities identified through the CART analysis. This provides a structured way to assess relative regional impacts on system adequacy, and helps to identify how disruptions in specific regions propagate throughout the system. Some regions may demonstrate wide variations in incremental impact depending on removals elsewhere in the system, while the impacts of removing other regions may be less sensitive to the broader system state. Identifying regions with impacts that are consistent across other system changes may be helpful for developing strategies to manage uncertainty and minimize overall system disruption.

## 3 Results

**Fig 3:**
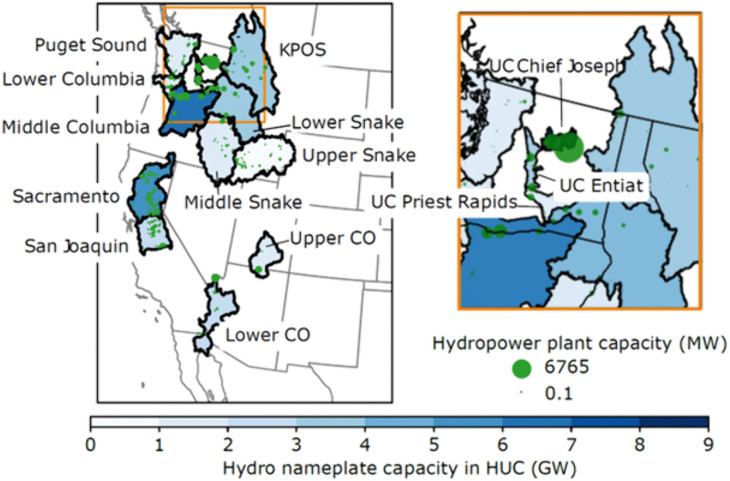
Resource adequacy as a function of capacity removal, across all 2^14^ hydropower availability scenarios considered.

We see that system resource adequacy levels vary widely across the range of hydropower availability scenarios considered, even when controlling for total capacity removal (Table 2). Fig 3 presents system NEUE under each of the 16,384 scenarios considered; higher levels of nameplate capacity removal are unsurprisingly correlated with higher levels of system risk, with the most extreme scenarios resulting in system NEUE on the order of 200 parts per million, about two orders of magnitude more risk than would be deemed acceptable by grid planners. However, we also see wide ranges of potential unserved energy for roughly similar levels of hydropower unavailability, indicating that the specifics of which resources are removed also plays an important role in determining overall system adequacy.

**Table 2:** Summary of min, median, and max EUE scenarios within 19.9–20.1 GW of hydropower unavailability.

Case	Hydrologic regions unavailable	Capacity unavailable (MW)	ΔEUE/MW (MWh)
Max EUE	Upper CO, Lower CO, KPOS, Middle Snake, Lower Columbia, UC Chief Joseph, UC Entiat, UC Priest Rapids	19,998	31.47
Median EUE	Upper CO, KPOS, Upper Snake, Middle Snake, Lower Snake, Middle Columbia, Lower Columbia, UC Entiat	20,060	4.27
Min EUE	Upper CO, Lower CO, Lower Snake, Middle Columbia, Lower Columbia, Puget Sound, Sacramento, San Joaquin	20,062	0.58

The rest of this section applies a series of analyses to understand these dynamics. First, we manually investigate the availability characteristics of a small set of scenarios that exhibit very different adequacy levels despite similar magnitudes of removal (Section 3.1). Next, we study the incremental reliability impact of hydropower unavailability in specific regions, given all possible conditions of hydropower unavailability in other regions (Section 3.2). We then apply CART analysis to quantify and rank average removal impacts across regions (Section 3.3). Finally, we consider how regional incremental removal impacts interact and change nonlinearly as hydropower becomes unavailable in the regions determined to be the most important (Section 3.4).

### 3.1 Case-based analysis of scenarios with comparable hydropower availability

To investigate why NEUE varies at similar levels of hydro capacity removal, we manually inspect the minimum, median, and maximum NEUE scenarios from the set of 179 cases with 19.9–20.1 GW removed. These cases represent very different adequacy outcomes (Table 2) but also very different spatial distributions of hydropower unavailability.

**Fig 4:**
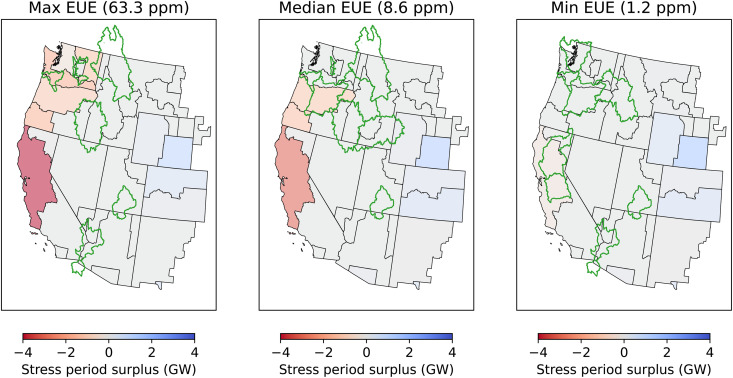
Average shortfall or surplus energy in each electrical balancing area at 21:00 on August 29 (weather year 2007) for the three scenarios described in Table 2. Green outlines indicate hydrologic regions that are unavailable in the corresponding scenarios.

In this initial exercise, we see that the scenario with the worst resource adequacy performance consists of hydropower removals clustered tightly around the Pacific Northwest watersheds and the two Desert Southwest HUCs (green outlines in Fig 4a), whereas in the other two cases the unavailable regions are distributed more diffusely, do not include the bigger Pacific Northwest watersheds, and, in the lowest EUE case, the Desert Southwest.

Fig 4 also compares the average energy deficit/surplus across these three representative scenarios for the highest-EUE hour in the maximum EUE scenario, providing insights into whether hydropower removal impacts are felt locally or are spread across the broader system. In the maximum EUE case, we see that hydropower losses in the Pacific Northwest drive high summer shortfall risk not just in nearby electrical balancing areas but also in California. This is also seen in the median case, where high levels of shortfall risk is observed in California, with some shortfall in the Pacific Northwest regions, despite most removals being out of state. Other regions across the interconnection either lack the surplus capacity to export or have surplus capacity but are unable to move it to regions with shortfall due to east-west transmission constraints. These regional, seasonal, and network interactions explain the variation in EUE across scenarios with similar levels of hydropower unavailability.

### 3.2 Visualizing variations in regional incremental impacts

**Fig 5:**
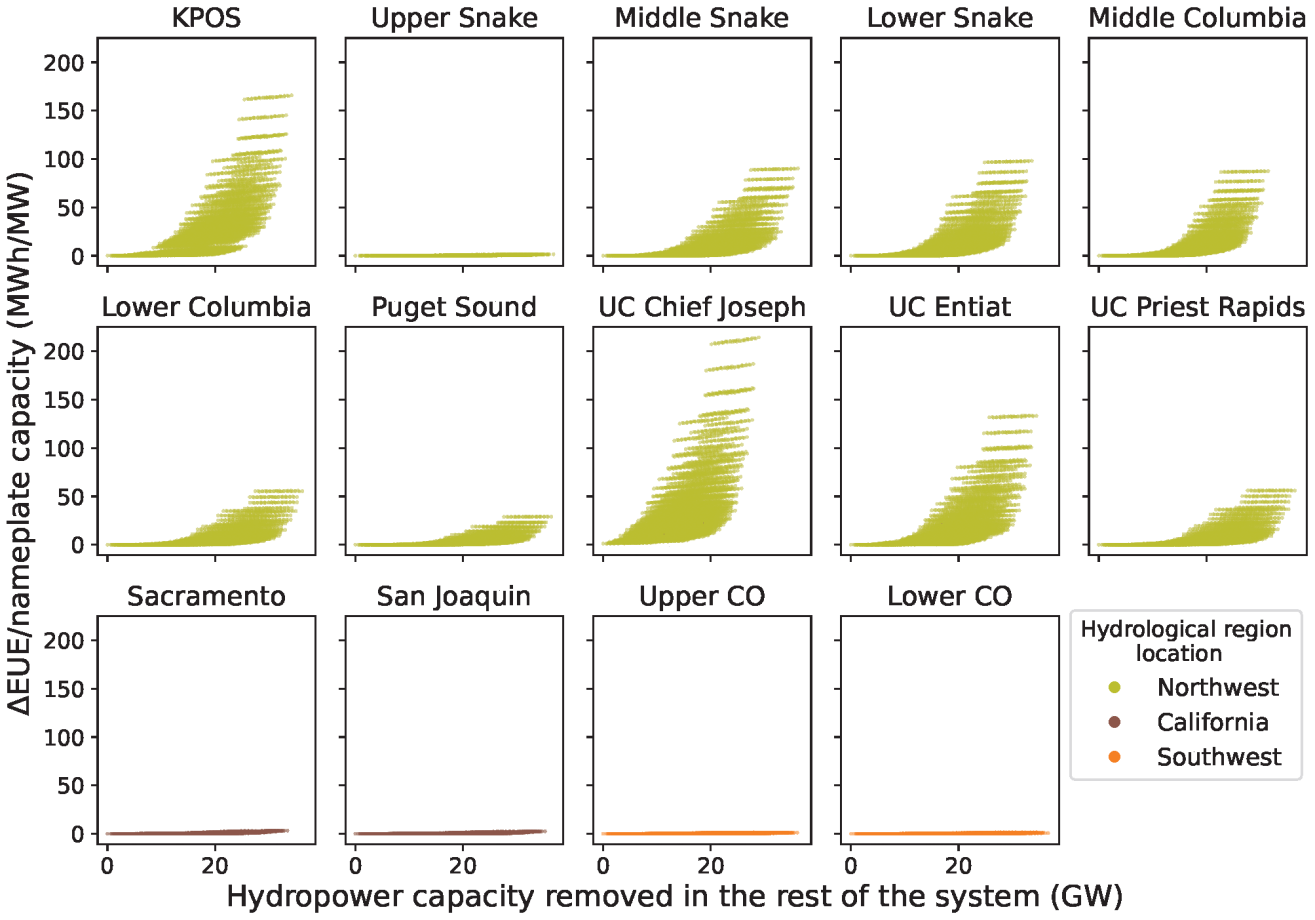
Capacity-adjusted incremental removal impact for each hydrological region, as a function of total hydropower capacity removed in the rest of the system. Each subplot visualizes incremental regional impacts under all possible states of hydropower availability.

Given the observed large variability in EUE for a given level of unavailable hydropower capacity, we next examine the incremental reliability impact of hydropower unavailability in specific regions. Marginal impacts on resource adequacy can differ depending on whether a region is rendered unavailable first or after other regions have also been impacted. To characterize this, we look at capacity-adjusted adequacy impacts between pairs of scenarios (change in EUE per unavailable capacity), where the only difference between the pair is a single hydrologic region’s unavailability. Fig 5 visualizes this incremental impact for all possible scenario pairs for each region.

These regional results reveal two key insights. First, impacts increase nonlinearly as more hydropower becomes unavailable elsewhere in the system, although the specifics of which regions are removed can still drive substantial variation in the magnitude of these impacts (visualized as vertical spread in the point clouds of Fig 5). We see hydropower loss tipping points, where incremental EUE impacts in a given region remain small up until a certain level of background hydropower unavailability, at which point they grow much more quickly as a function of prior removals. This is consistent with Fig 3, which showed that increases to overall system NEUE remain small up until threshold hydropower removal levels are surpassed.

The second insight is that these tipping point thresholds, as well as the rate of growth of incremental impacts once they are surpassed, vary widely across different regions. In some regions, these thresholds are not surpassed at all. For example, losing the Desert Southwest hydrologic regions (Upper and Lower Colorado) results in minimal incremental adequacy impacts, even when substantial hydropower capacity has been removed elsewhere in the system. The Upper Snake, Sacramento, and San Joaquin regions only show a limited increase in incremental impact under higher removal levels elsewhere in the system, while impacts elsewhere in the Pacific Northwest are much more sensitive to the state of the rest of the system once their threshold has been surpassed. UC Chief Joseph, which contains the Grand Coulee and Chief Joseph dams and has the most installed capacity out of any region considered, has a much lower threshold than the rest of the regions, reflecting that it is almost able to “self-tip” and single-handedly drive the entire system into shortfall conditions.

### 3.3 Important regions from sequential screening

**Fig 6:**
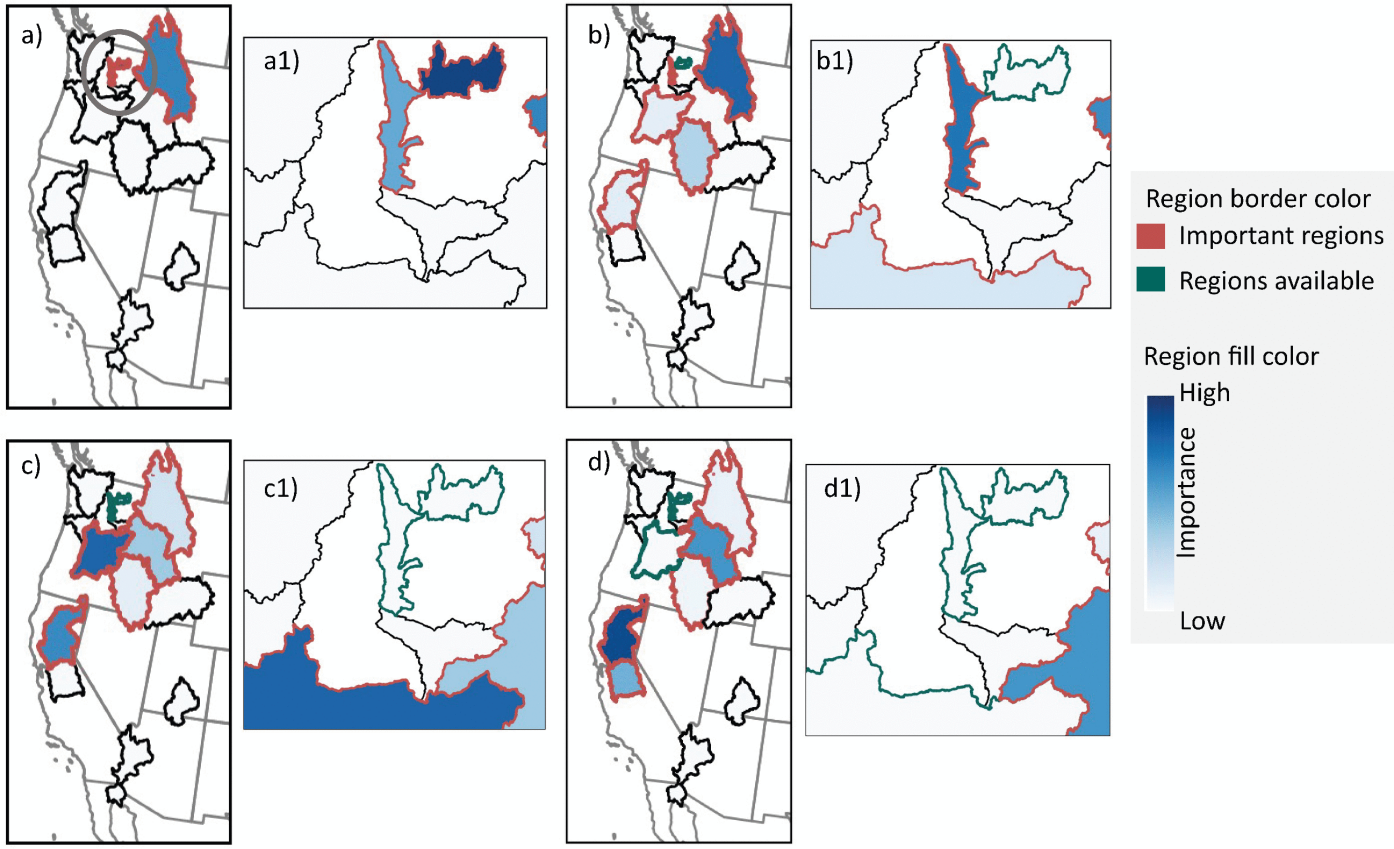
Importance of regions identified from CART analysis using the Gini importance score for a) all scenarios, b) selected scenarios with retention of UC Chief Joseph, c) selected scenarios with retention of UC Chief Joseph and UC Entiat regions, d) selected scenarios with retention of UC Chief Joseph, UC Entiat and Middle Columbia regions. The corresponding subplots a1, b1, c1, and d1 are the zoomed maps as highlighted by the circle in subplot a. The red border represents important regions, and green represents regions where hydropower plants are held to be always available. The influence of each region is shown by shading ranging from white (low importance) to light blue (moderate importance) and dark blue (high importance).

The previous section offers initial insights into which hydropower regions are the most important for resource adequacy but does not rigorously quantify their relative importance under deep uncertainty. Sequential screening CART analysis (as described in Section 2.4) is thus used to do so and to demonstrate how relative importance changes after setting aside, or screening, regions that are initially determined as the most impactful among the full set of hydrological regions. The process of identifying important regions from sequential screening clarifies influential regions beyond the most obvious high-capacity region (UC Chief Joseph). Fig 6 illustrates the spatial distribution of these critical regions, their importance being quantified by the Gini metric used in the CART analysis. The red borders mark highly influential regions (with Gini importance score greater than zero), identified through scenario discovery, while green borders represent regions that remain available or are retained through the screening process. Regions with a black border have negligible contribution in the splitting of the CART tree. For all exploratory scenarios, we identify UC Chief Joseph, KPOS, and UC Entiat as the most influential regions, ranked in decreasing order of importance (The colored regions in Fig 6a, 6a1). This result reflects the first iteration in the sequential screening process. UC Chief Joseph emerges as an expected influential region due to its high nameplate capacity compared to other regions. UC Chief Joseph’s importance is also driven by its proximity to the Seattle load center. While this plant is treated as a non-dispatchable run-of-river facility, its generation profile aligns with power system needs during stress periods, and its value during these periods could be amplified when additional hydropower capacity with a similar profile is lost because the local grid is largely dominated by hydropower resources.

We would expect Middle Columbia and Sacramento to have high importance due to their hydropower capacities ranking second and third (Table 1); with Chief Joseph as the highest. Instead, KPOS and UC Entiat exhibit strong influence, indicating the importance of their interconnections with other regions and their role in energy distribution across the system, and low generation capacity of Sacramento.

In the second iteration of the sequential screening process, we only consider scenarios where UC Chief Joseph remains available due to its highest importance in the first iteration. This step aids in identifying additional influential regions beyond the highest-capacity one. For the second iteration of the CART analysis, as we exclude the scenarios where UC Chief Joseph is unavailable, the next most influential regions identified are KPOS, Middle Snake, UC Entiat, Sacramento, and Middle Columbia (Fig 6b, 6b1). Among these, UC Entiat and KPOS were previously identified, proving their continued influence on the system, with UC Entiat having the highest importance. So, UC Chief Joseph and UC Entiat are kept available for the next round of screening. In the third iteration of the CART analysis, we identify KPOS, Lower Snake, Middle Snake, Sacramento, and Middle Columbia (which has the highest importance) as important regions (Fig 6c, 6c1), where Lower Snake is a new addition to the set of important regions compared to the previous iteration. In the last round of iteration, CART analysis is performed for the subset of scenarios with UC Chief Joseph, UC Entiat, and Middle Columbia regions always available. A new region that emerges as important is San Joaquin which is now an additional region impacting system performance. The emergence of new influential regions across different iterations (e.g., Lower Snake, San Joaquin) highlights the regional dependencies of their availability.

### 3.4 Identifying high- and low-impact regions

**Fig 7:**
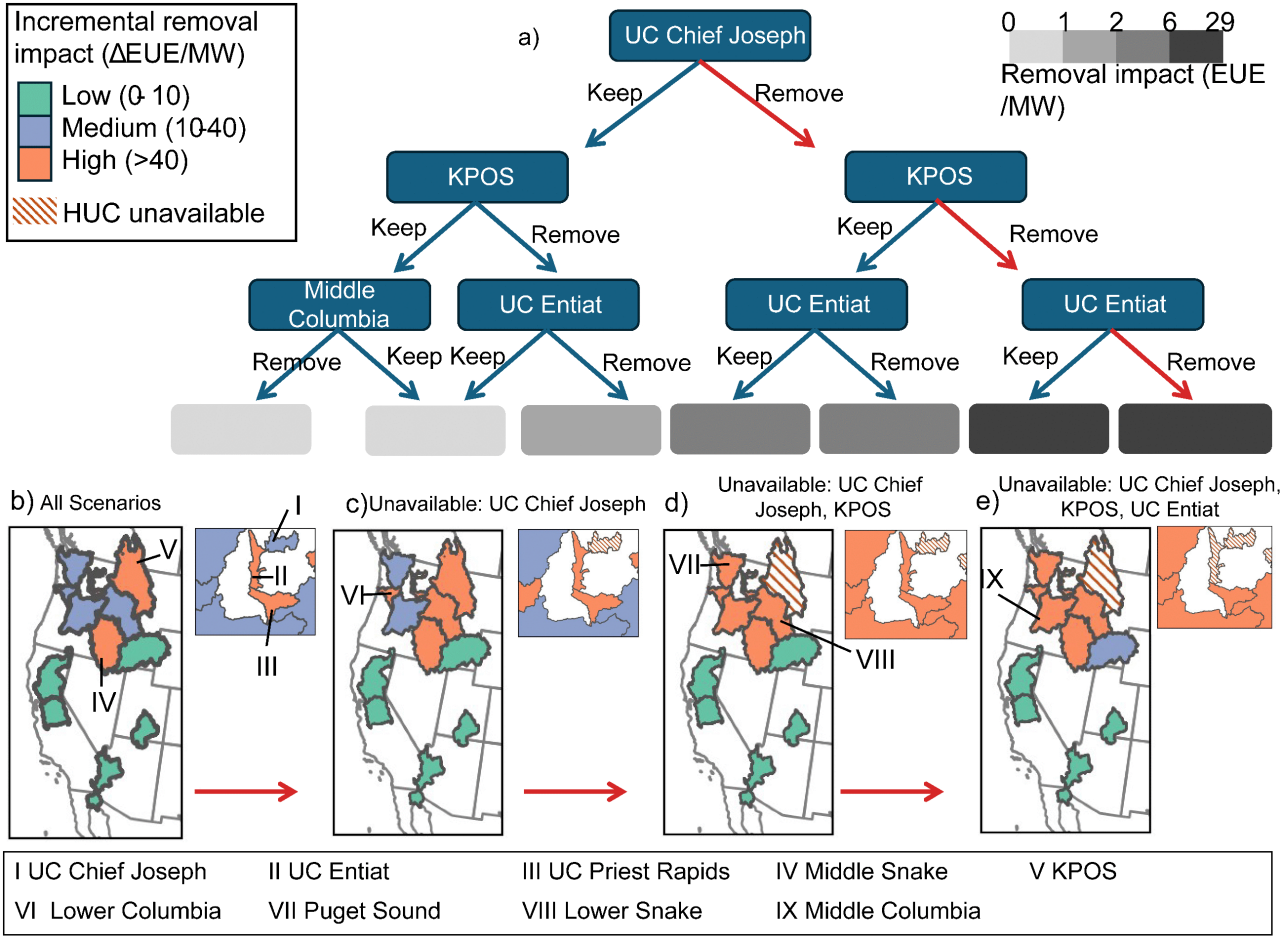
(a) The tree obtained from the CART analysis of all scenarios, with expected unserved energy (EUE) per megawatt of capacity as the criterion. The branching structure represents the availability of regions, where “Keep"” indicates that the scenarios include the availability of the region and “Remove"” signifies that the region is unavailable in the scenarios. At the leaf nodes, gray-shaded boxes represent EUE per megawatt of capacity, with darker shades indicating higher values, as shown in the legend. (b, c, d, e) The subplots illustrate the spatial variation of removal impacts for a selected branch of the tree, highlighted by red arrows. The color of each region represents its classification based on its average incremental impact on system EUE per megawatt of capacity removed. The hatch pattern indicates regions that are unavailable across all considered scenarios.

The CART analysis can be visually represented as a series of keep-or-remove experimental decisions to explore the region availability scenarios and their impacts on EUE per megawatt of capacity (Fig 7a). As we navigate along the tree, the sequence determines how region unavailability influences system performance (EUE per megawatt of capacity). Each branch corresponds to a decision rule based on region availability. Each node represents the binary decision of whether the region is available (Keep) or unavailable (Remove). The end nodes (leaves) show the resulting EUE per megawatt of capacity, with darker shading indicating higher average impact. This tree provides a structured decision framework to prioritize regions in system adequacy planning. The regions not present on the tree do not significantly impact system performance when unavailable. In the tree, starting at the root node, all scenarios are included. The first split occurs at UC Chief Joseph, indicating it has the highest importance in system performance (Fig 6a). This tree is obtained from the first iteration of sequential screening (Section 3.3; Fig 6a). The other three trees from the sequential screening process are included in the supplementary material (S2–S4 Figs).

To visually analyze spatial dependencies between regions, we select a specific branch of the tree, highlighted by red arrows, and examine the corresponding spatial patterns of incremental resource adequacy impacts in Fig 7 (b-e). The color of each region represents its classification based on the difference in EUE per megawatt of capacity between scenarios with and without hydropower in the corresponding region, categorized into three levels of impact: low (0–10), medium (10–40), and high (>40). In Fig 7b, which represents the root node where all scenarios are included, the most consequential regions are UC Entiat, UC Priest Rapids, KPOS, and Middle Snake, all of which fall into the high-impact category (EUE/MW capacity difference > 40). This indicates that their unavailability significantly deteriorates system performance across different scenarios. As we navigate along the tree, Fig 7c focuses on a subset of scenarios where UC Chief Joseph is unavailable, revealing Lower Columbia as an additional consequential region, suggesting a dependency between UC Chief Joseph’s availability and Lower Columbia’s contributions. Further along the branch, when both UC Chief Joseph and KPOS are unavailable, Puget Sound and Lower Snake emerge as consequential regions. Toward the end of the branch, when UC Chief Joseph, KPOS, and UC Entiat are all unavailable, Middle Columbia is also identified as a consequential region. These results highlight the path-dependent effects of a region’s availability, demonstrating how the availability of certain regions emphasizes the interconnections within the system.

We also identify robust regions as those that consistently demonstrate low-adequacy impacts across different scenarios, indicating that their hydropower unavailability does not significantly deteriorate system performance, regardless of the availability of other resources. These regions include Upper Colorado Dirty Devil, Lower Colorado, San Joaquin, and Sacramento, as they consistently remain in the low-impact category throughout the analysis (Fig 7b-7e). Additionally, Upper Snake is found to be high impact only in the subset of scenarios when UC Chief Joseph, KPOS and UC Entiat is unavailable (Fig 7d). This suggests that while these regions generally maintain adequacy, their performance impact is contingent upon the availability of other regions. Identifying these regions helps differentiate between regions that are more or less consequential to resource adequacy, even under varying conditions of hydropower unavailability in other regions.

## 4 Discussion and conclusion

Our results show that losses in hydropower availability can have complex, interactive effects on resource adequacy in the Western Interconnection. Intuitively, resource adequacy impacts increase with increasing hydropower unavailability. However, this relationship is nonlinear and region-specific, with the largest incremental impacts being associated with the Columbia River basin and the smallest with the Colorado River basin. While the Pacific Northwest, which has the greatest concentration of hydropower in the US, often has the most consequential hydropower for driving resource adequacy outcomes, unserved energy is often experienced in other regions such as California. Meanwhile, hydropower in certain regions such as the Desert Southwest has little impact on resource adequacy outcomes in the West. Unserved energy is also observed when some regions have surplus energy, indicating that transmission expansion could improve resource adequacy under conditions with less hydropower.

The sequential screening process to identify influential regions highlights the regional dependencies and interaction effects that determine the grid adequacy impact of hydropower unavailability. CART analysis allows us to quantify the relative importance of individual regions in maintaining adequacy of the studied system and confirms the most consequential regions are in the Columbia River basin while the least consequential are in the Colorado River basin. However, we also demonstrate that additional regions become more impactful under specific conditions; for example, scenarios that keep UC Chief Joseph, UC Entiat, and Middle Columbia online reveal the importance of the Lower Colorado, San Joaquin, and Lower Snake basins under those conditions. Meanwhile, some regions demonstrate limited loss impacts across many unavailability scenarios, indicating system-level insensitivity to hydropower outcomes in these regions.

While the importance of Pacific Northwest hydropower locally and to California is well-known, this analysis provides a thorough, robust assessment that quantifies which hydropower regions affect each other and by how much. It demonstrates that different regions are more or less important under different conditions of hydropower unavailability, highlighting the importance of understanding complex interregional relationships when considering the broader implications of reduced hydropower availability. This complexity would only increase with additional scenario dimensions involving changes to energy and water systems and the human and natural processes that influence them.

Thus, future research could focus on assessing the impacts of hydropower unavailability across various scenarios of future infrastructure, climate conditions, or other uncertain power sector outcomes to better understand the vulnerabilities of the Western Interconnection [51,52]. Variations in precipitation patterns, temperature, and extreme weather events can significantly affect hydropower generation, leading to potential energy shortages and reliability concerns. Additionally, integrating socioeconomic factors, such as policy interventions and market adaptations, would contribute to understanding robust regions in the system. For example, the One Big Beautiful Bill Act of 2025 removes tax incentives for wind and solar technologies. Scenarios including this policy might have higher shares of other technologies such as natural gas that provide firm capacity and affect the relative contribution of hydropower towards resource adequacy requirements. Further, exploring alternative energy storage solutions, adaptive infrastructure designs, and transboundary water management approaches could also be crucial to mitigating the risks associated with hydropower dependency under deep uncertainties. In our analysis, the relative importance of regional hydropower unavailability is derived from considering system resource adequacy. However, hydropower plants provide other critical services to the grid, including load balancing, frequency stabilization, and black start capabilities. Future analyses can consider these services as well.

In this study, scenario design follows a Decision Making Under Deep Uncertainty (DMDU) framework, which emphasizes exploration of broad ensembles of possible futures rather than assigning predictive probabilities. The goal is to characterize the envelope of consequential outcomes and identify system vulnerabilities across a wide range of conditions, with scenarios evaluated a posteriori using expert judgment to assess relevance and feasibility. Because there is no empirically grounded basis for assigning defensible prior probabilities to many of the disruptions considered beyond the general understanding that complete regional hydropower loss is unlikely imposing probability weights would be inherently subjective and could limit the exploratory scope of the analysis. Accordingly, highly extreme scenarios are included not as forecasts, but as bounding stress tests to probe system sensitivity and identify potential vulnerabilities; their plausibility and policy relevance are therefore discussed qualitatively.

A key limitation of the present analysis is the assumption of full capacity hydropower unavailability, which represents a simplified and intentionally conservative abstraction of real-world conditions. In practice, hydropower constraints are often partial, time-varying, and shaped by ecological flow requirements, reservoir management strategies, and operational flexibility. Future work will therefore explore time-varying and partial-availability representations to more realistically capture ecological and operational constraints and better reflect the dynamic nature of hydropower system performance.

Future work could therefore consider additional reservoir operations and water management considerations that impact the flexibility of the hydropower fleet to respond to lost power production at one or more facilities. Doing so could more realistically capture ecological and other operational constraints and better reflect the complex nature of hydropower system operation. For example, our analysis is constrained by the capabilities of the resource adequacy model, so it cannot capture any shifting of hydropower energy budgets across seasons. Developing a framework that allows some degree of long-term hydropower planning and more closely represents the complete set of hydropower operating constraints would improve the realism of system operations and provide a more accurate assessment of reliability impacts.

Our methodology described in this work is also extensible to natural gas plants and other technologies and projects [53]. While this analysis focuses on exploratory modeling of hydropower unavailability, we could extend it to similar common-mode failures in gas or other connected generation devices. The general procedures implemented herein could also be expanded to include other metrics and incorporate different or additional models and analysis tools. While this work contributes specifically to the conversation about hydropower value for resource adequacy, it could provide a template for applying scenario discovery approaches with applied systems models to inform a broad range of energy-water discussions and beyond.

## Supporting information

S1 Fig Distribution of combinations of hydropower unavailability scenarios as a function of nameplate capacity unavailable. (PNG)

S2 Fig The tree obtained from the analysis for selected scenarios with UC Chief Joseph available. The branching structure represents the availability of regions, where “Keep” indicates that the scenarios include the availability of the region and “Remove” signifies that the region is unavailable in the scenarios. At the leaf nodes, grey-shaded boxes represent EUE per MW capacity, with darker shades indicating higher values, as shown in the legend.
(PNG)

S3 Fig The tree obtained from the analysis for selected scenarios with UC Chief Joseph and UC Entiat available. The branching structure represents the availability of regions, where “Keep” indicates that the scenarios include the availability of the region and “Remove” signifies that the region is unavailable in the scenarios. At the leaf nodes, grey-shaded boxes represent EUE per MW capacity, with darker shades indicating higher values, as shown in the legend.
(PNG)

S4 Fig The tree obtained from the analysis for selected scenarios with UC Chief Joseph, UC Entiat and Middle Columbia available. The branching structure represents the availability of regions, where “Keep” indicates that the scenarios include the availability of the region and “Remove” signifies that the region is unavailable in the scenarios. At the leaf nodes, grey-shaded boxes represent EUE per MW capacity, with darker shades indicating higher values, as shown in the legend.
(PNG)

S5 Fig Monthly regional load variation across different p-regions. (PNG)

S6 Fig Monthly solar generation variation across different p-regions. (PNG)

S7 Fig Monthly wind generation variation across different p-regions. (PNG)
