# Supplementary figures and images for: Impact of hydropower availability on resource adequacy of the United States western interconnection
Source: PLoS One. 2026 Jun 23;21(6):e0351321. doi: 10.1371/journal.pone.0351321 (PMC13289947; doi:10.1371/journal.pone.0351321)

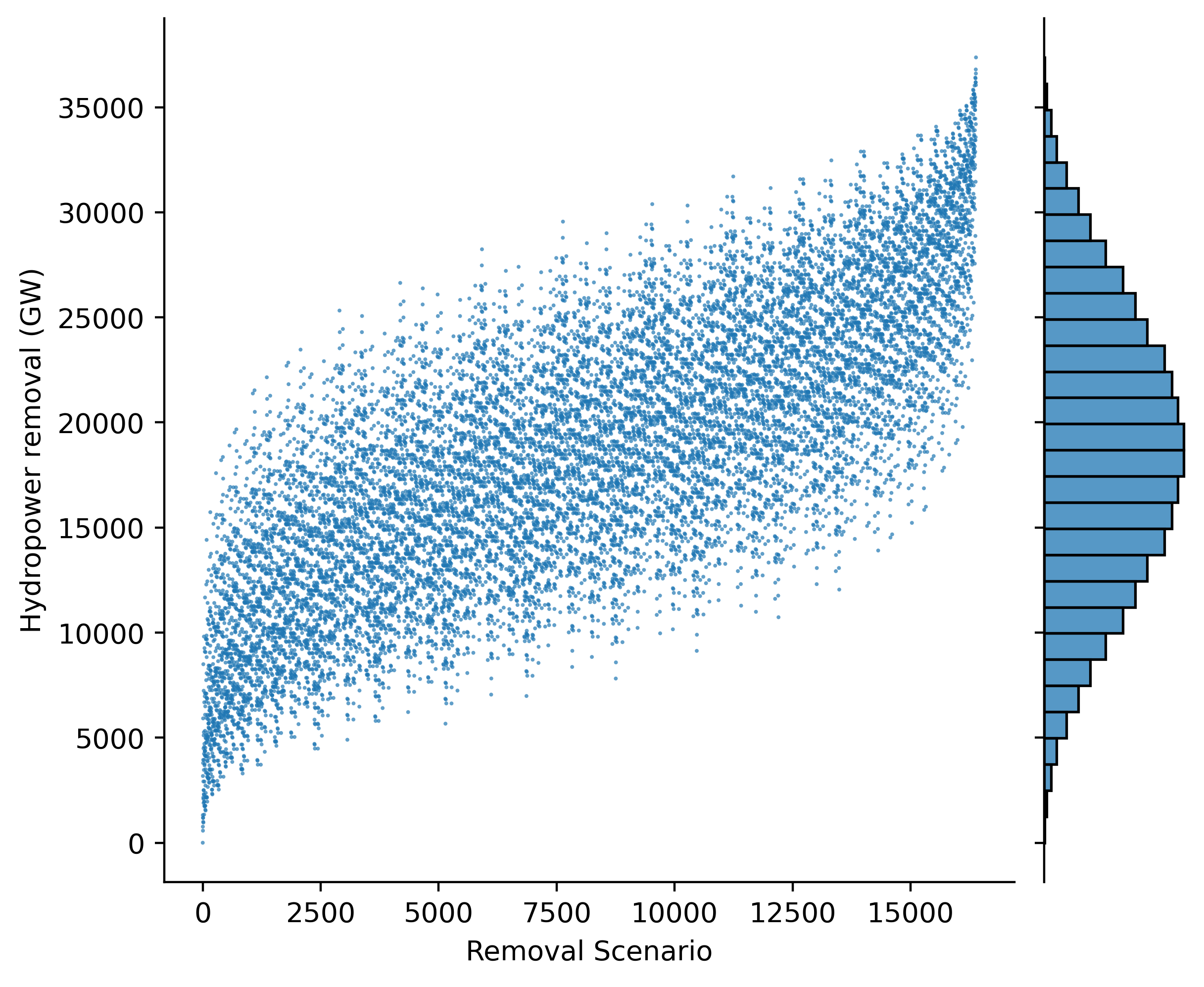

Supplement: S1 Fig — (PNG) [file pone.0351321.s001.png]

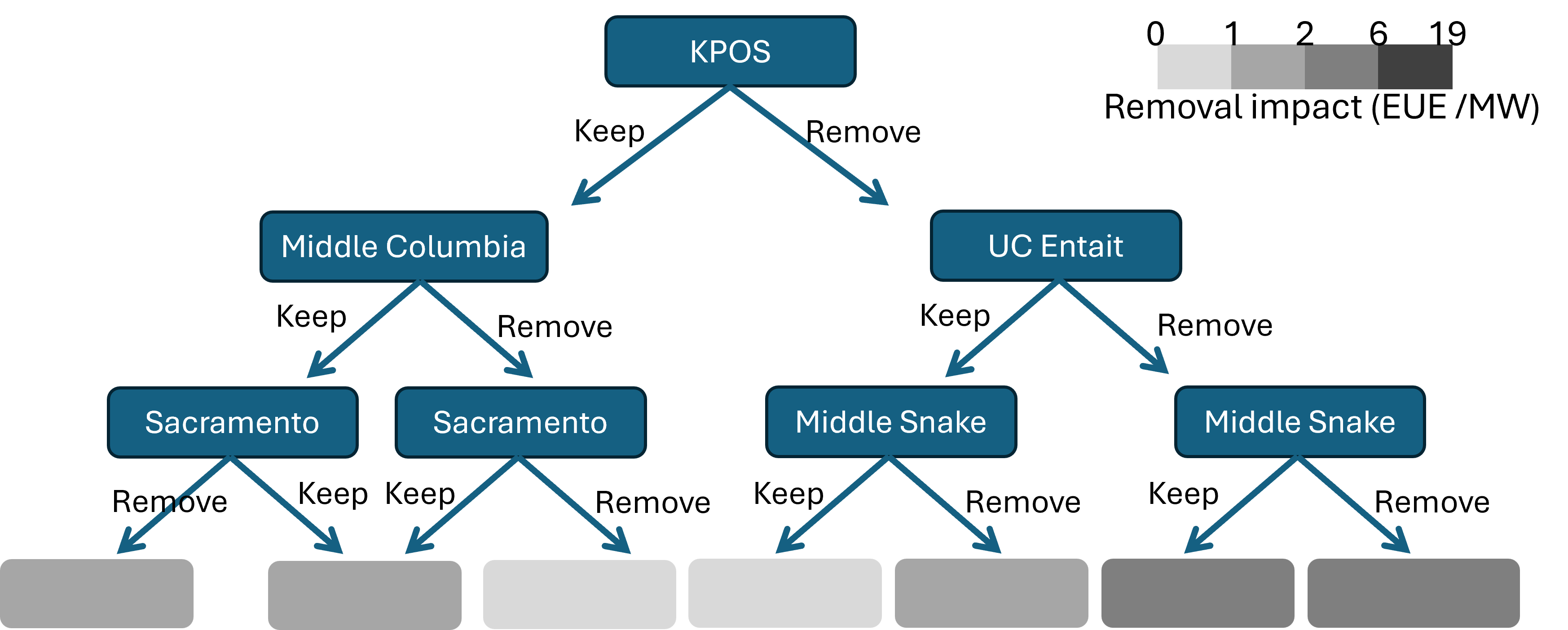

Supplement: S2 Fig — The branching structure represents the availability of regions, where “Keep” indicates that the scenarios include the availability of the region and “Remove” signifies that the region is unavailable in the scenarios. At the leaf nodes, grey-shaded boxes represent EUE per MW capacity, with darker shades indicating higher values, as shown in the legend. (PNG) [file pone.0351321.s002.png]

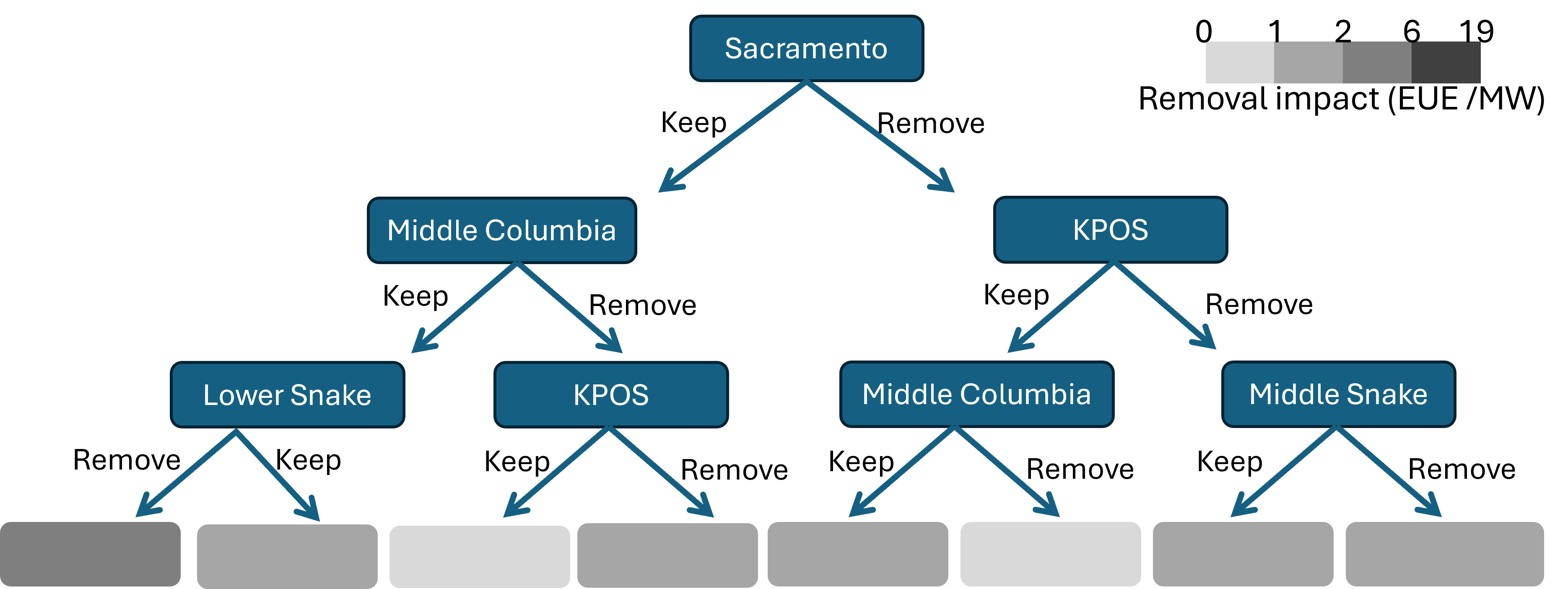

Supplement: S3 Fig — The branching structure represents the availability of regions, where “Keep” indicates that the scenarios include the availability of the region and “Remove” signifies that the region is unavailable in the scenarios. At the leaf nodes, grey-shaded boxes represent EUE per MW capacity, with darker shades indicating higher values, as shown in the legend. (PNG) [file pone.0351321.s003.png]

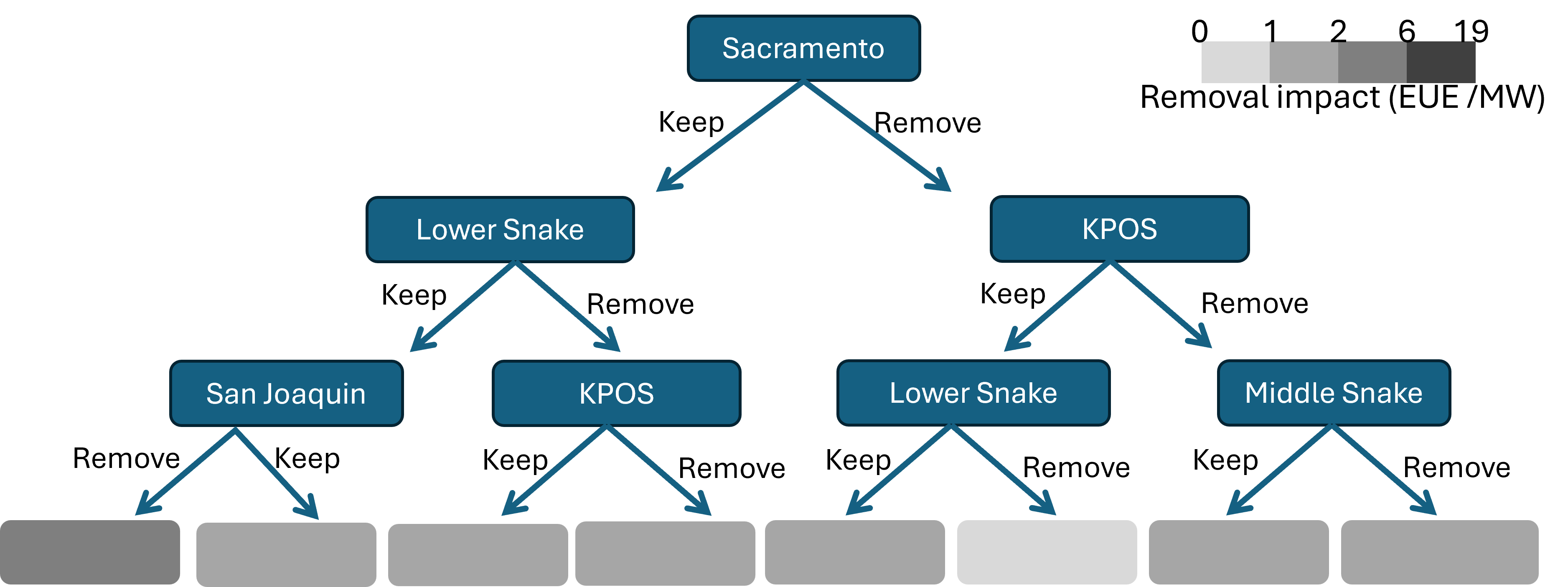

Supplement: S4 Fig — The branching structure represents the availability of regions, where “Keep” indicates that the scenarios include the availability of the region and “Remove” signifies that the region is unavailable in the scenarios. At the leaf nodes, grey-shaded boxes represent EUE per MW capacity, with darker shades indicating higher values, as shown in the legend. (PNG) [file pone.0351321.s004.png]

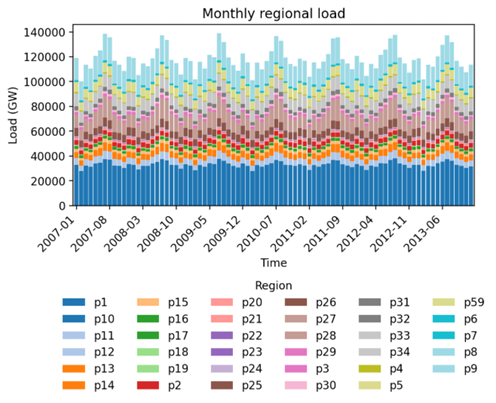

Supplement: S5 Fig — (PNG) [file pone.0351321.s005.png]

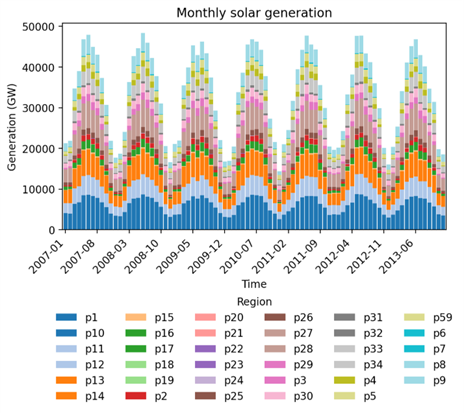

Supplement: S6 Fig — (PNG) [file pone.0351321.s006.png]

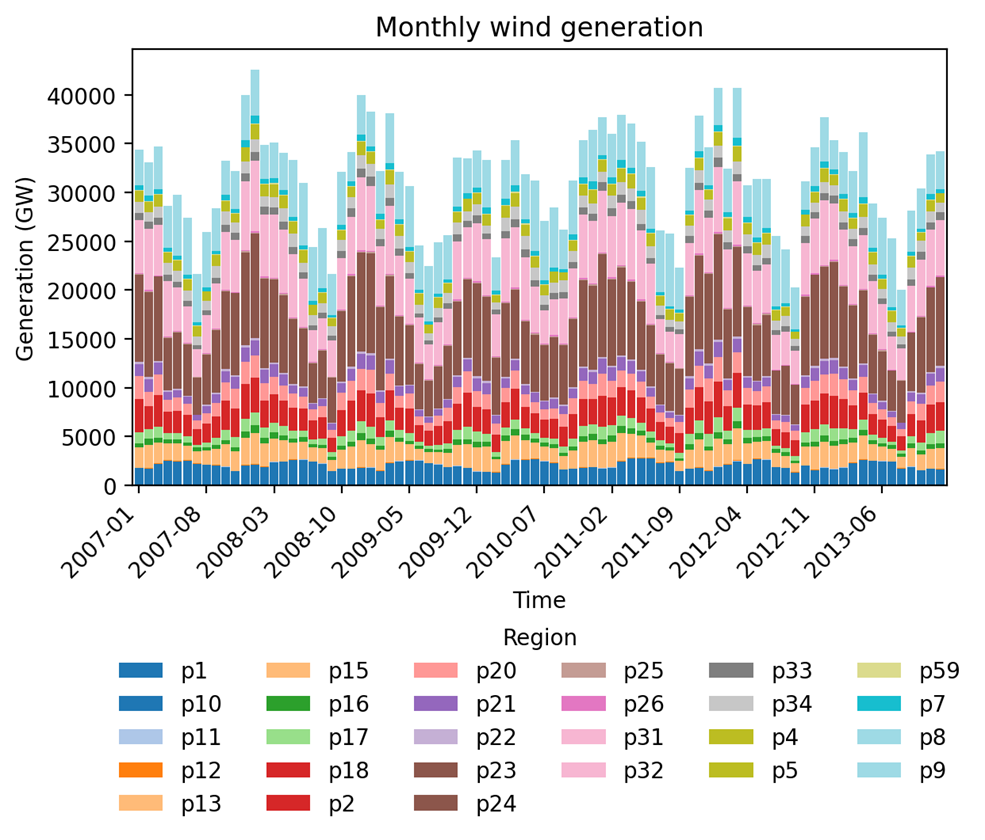

Supplement: S7 Fig — (PNG) [file pone.0351321.s007.png]
